# Quantification of blood-brain barrier permeability by dynamic contrast-enhanced NIRS

**DOI:** 10.1038/s41598-017-01922-x

**Published:** 2017-05-10

**Authors:** Daniel Milej, Androu Abdalmalak, Lise Desjardins, Hassaan Ahmed, Ting-Yim Lee, Mamadou Diop, Keith St. Lawrence

**Affiliations:** 1 Imaging Division, Lawson Health Research Institute, London, ON Canada; 2 Department of Medical Biophysics, Western University, London, ON Canada; 3 Imaging Research Laboratories, Robarts Research Institute, London, ON Canada

## Abstract

The blood-brain barrier (BBB) is integral to maintaining a suitable microenvironment for neurons to function properly. Despite its importance, there are no bedside methods of assessing BBB disruption to help guide management of critical-care patients. The aim of this study was to demonstrate that dynamic contrast-enhanced (DCE) near-infrared spectroscopy (NIRS) can quantify the permeability surface-area product (PS) of the BBB. Experiments were conducted in rats in which the BBB was opened by image-guided focused ultrasound. DCE-NIRS data were acquired with two dyes of different molecular weight, indocyanine green (ICG, 67 kDa) and 800CW carboxylate (IRDye, 1166 Da), and PS maps were generated by DCE computer tomography (CT) for comparison. Both dyes showed a strong correlation between measured PS values and sonication power (R^2^ = 0.95 and 0.92 for ICG and IRDye respectively), and the PS values for IRDye were in good agreement with CT values obtained with a contrast agent of similar molecular weight. These proof-of-principle experiments demonstrate that DCE NIRS can quantify BBB permeability. The next step in translating this method to critical care practice will be to adapt depth sensitive methods to minimize the effects of scalp contamination on NIRS PS values.

## Introduction

The blood-brain barrier (BBB), which consists of tight junctions between adjacent endothelial cells, is essential to brain homeostasis as it limits the passage of molecules from blood to brain parenchyma^1, 2^. Increased permeability resulting from BBB dysfunction has been reported following traumatic brain injury^3^, stroke and subarachnoid hemorrhage^4^. Breakdown of the barrier is believed to contribute to secondary brain injury by allowing white blood cells to enter the brain, contributing to neuroinflammation, disrupting proper regulation of ionic and molecular fluxes, and leading to vascular edema^5^. It may also contribute to delayed cerebral ischemia as activated monocytes can release the potent vasoconstrictor, endothelin^−1^. Clinically, increased levels of proinflammatory cytokines in the cerebrospinal fluid are associated with worse outcome^5^. However, the limited availability of methods that can detect BBB disruption (BBBD) has hindered this area of investigation.

Since the intact BBB restricts the entrance into the brain to small (<400 Da) lipophilic molecules, an established method of assessing barrier disruption is by detecting the retention of a larger molecule, such as a contrast agent, in the interstitial space. The feasibility of *in vivo* monitoring of BBB permeability using optical contrast agents was first presented in a mouse stroke model^6^. A similar approach was developed for near-infrared spectroscopy (NIRS) using the clinically approved contrast agent Indocyanine Green (ICG), and it was shown that uptake in the brain was greater following Mannitol-induced disruption^7^. However, this is a relatively slow procedure, requiring ICG measurements up to 40 min post injection and it does not provide an estimate of BBB permeability. Dynamic contrast-enhanced (DCE) approaches, which are well established in imaging studies involving computed tomography (CT) and magnetic resonance imaging (MRI), are an attractive alternative since data acquisition is typically completed in less than 10 min and permeability can be quantified using a tracer kinetic model to characterize contrast agent clearance^8, 9^.

Dynamic contrast-enhanced methods have been developed for NIRS, primarily for assessing cerebral blood flow (CBF)^10^. By incorporating depth-discriminating methods such as time-resolved (TR)^11, 12^ detection to separate extra- and intracerebral signal contributions, DCE NIRS has been shown to be sensitive to perfusion changes associated with cerebral ischemia and to provide quantitative CBF measurements^13–16^. In a recent study, Liebert *et al*. observed significant differences in the clearance rate of ICG from the brains of patients with known BBB disruption compared to healthy controls – a strong indication that DCE NIRS is sensitive to BBB permeability^17^. However, other factors can influence the shape of DCE curves, namely the rate of dye delivery and cerebral hemodynamics. Furthermore, this qualitative approach provides no means of assessing the degree of BBB permeability, which is related to the risk of vasogenic edema^18^.

In this study we present a DCE NIRS method of measuring the permeability surface-area (PS) product based on a kinetic modelling approach used previously to characterize vascular leakage in tumors^19^. To investigate the sensitivity of DCE NIRS to changes in BBB permeability, experiments were conducted in rats in which the BBB was opened by image-guided focused ultrasound (FUS)^20, 21^. This approach enabled the location of BBB opening to be positioned in the sensitivity volume of the NIRS probes and the degree of permeability to be varied by adjusting the FUS power^22^. To further assess the sensitivity of the method, experiments were conducted using two optical contrast agents of different molecular weights since permeability is inversely related to the size of the agent^23^. Experiments were conducted using ICG (Sigma-Aldrich, Saint Louis, MO, US), which has a molecular weight of 67 kDa due to binding with albumin, and IRDye 800 carboxylate (LI-COR Biosciences, Lincoln, NE, US), which weighs 1166 Da. For validation, the PS product was measured independently by DCE CT^24^.

## Results

**Figure 1:**
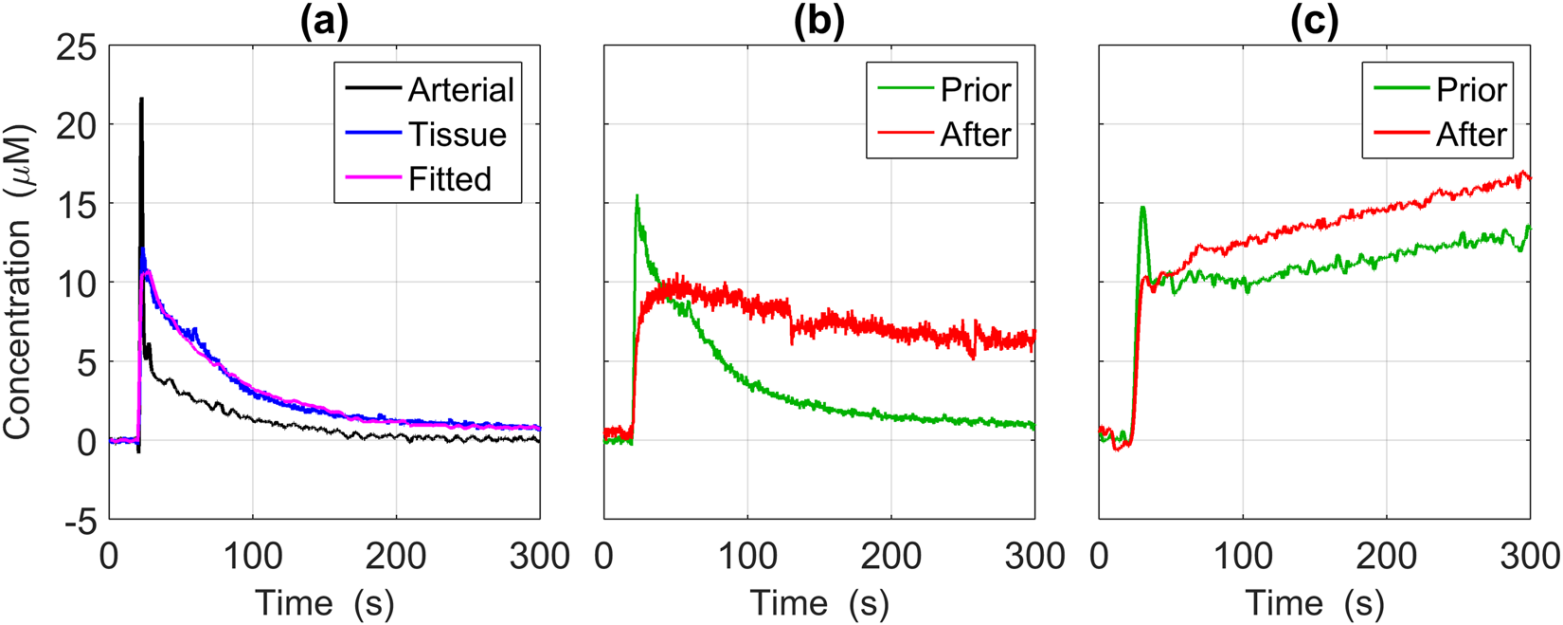
(**a**) Representative tissue ICG (blue) and arterial blood (black) ICG curves from one experiment prior to sonication. The best fit of the kinetic model to the ICG tissue concentration curve is also presented (magenta). Representative tissue concentration curves prior to and after sonication for ICG and IRDye are illustrated in (**b**) and (**c**) respectively. For illustration purposes, all tissue concentration curves were scaled by a factor of 30. The slower clearance of IRDye compared to ICG is due to their different molecular weights.

Data were obtained from eight rats (weight = 480 ± 120 g), which were divided equally into two groups based on the optical contrast agent used. Two measurements, one prior to and the other after sonication, were obtained for each rat. Figure 1a presents representative arterial and tissue concentration curves from one experiment involving ICG. Also shown is the best fit of the kinetic model. Figure 1b and c present brain concentration curves before and after sonication (FUS_POWER_ = 2 W) for ICG and IRDye, respectively. In the ICG example, the dye clearance was considerably slower post sonication, reflecting the extravasation of the dye due to the opening of the BBB. This difference was evident in the change in the PS product measured before (~0 ml/100 g/min) and after sonication (2.93 ml/100 g/min). Due to its small molecular weight, evidence of extravasation was evident for IRDye even at baseline (PS = 2 ml/100 g/min); however, permeability still increased after sonication (PS = 15 ml/100 g/min).

**Figure 2:**
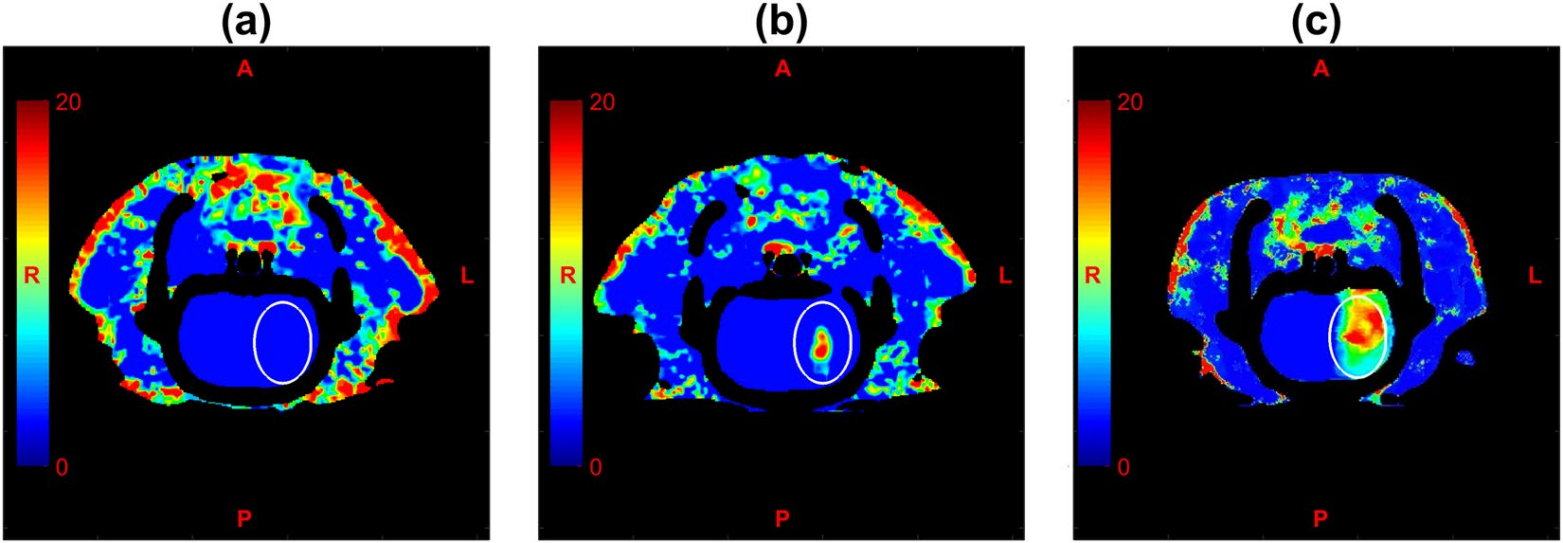
Maps of the permeability surface-area product (PS) generated by CT perfusion software: (**a**) prior to sonication, (**b**) after sonication, FUS_POWER_ = 1 W, same animal as in (**a**), and (**c**) after sonication FUS_POWER_ = 2 W, different animal. The oval represents the ROI used in CT data analysis (see Methods).

In each experiment, BBB permeability was measured independently by CT. Representative maps of the PS product are shown in Fig. 2: (a) prior to sonication, (b) same animal, after sonication (FUS_POWER_ = 1 W), and (c) different animal after sonication (FUS_POWER_ = 2 W). The integrity of the BBB is evident in Fig. 2a by the cool color throughout the brain, corresponding to a mean PS of 1.06 ± 0.24 ml/100 g/min. In the post sonication images, the focal increase in BBB permeability is clearly seen by the high intensity region in the left hemisphere, which was the hemisphere interrogated by NIRS. In general, the area of BBB disruption increased with FUS power. In the permeability map shown in Fig. 2c, which corresponded to the maximum FUS power, greater PS values were found throughout the left hemisphere.

A summary of PS and CBF values, pre and post sonication, for the two optical dyes and CT is given in Table 1. Although the mean PS product for IRDye at baseline was greater than zero, this difference did not reach statistical significance. Analysis of the blood flow results indicated a significant reduction after sonication; however, no difference was found between the NIRS and CT CBF measurements.

**Table 1:** Mean pre and post sonication CBF and PS values obtained by DCE NIRS and CT as well as individual PS values measured at each of the four sonication powers.

		Pre sonication		Post Sonication				
		$\overline{{\bf{CBF}}}$ (ml/100 g/min)	$\overline{{\bf{PS}}}$ (ml/100 g/min)	$\overline{{\bf{CBF}}}$ (ml/100 g/min)	PS (ml/100 g/min)			
					0.5 W	1 W	1.5 W	2 W
ICG	DCE NIRS	85.3 ± 22.4	0.06 ± 0.13	72.1 ± 18.5	0.41	1.09	1.63	2.93
	CT	70.3 ± 0.63	1.06 ± 0.24	55.8 ± 2.17	3.96	6.15	11.20	14.56
IRDye	DCE NIRS	71.8 ± 6.6	1.94 ± 1.36	58.8 ± 5.8	3.19	4.89	11.27	15.15
	CT	74.5 ± 5.83	1.08 ± 0.32	65.7 ± 26.5	3.64	7.25	9.98	16.97

**Figure 3:**
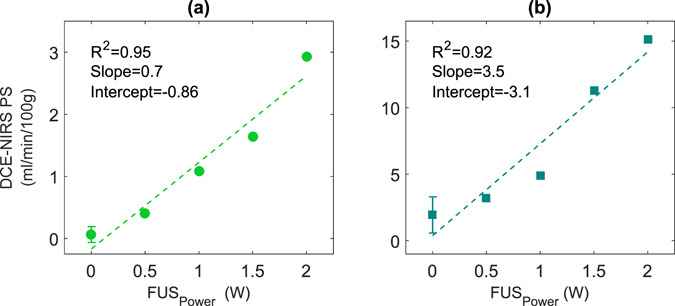
Permeability surface-area (PS) values obtained for ICG and (**b**) for IRDye at different sonication powers. Higher leakage of IRDye reflects its smaller size, resulting in increasing permeability. The error bars at FUS_Power_ = 0 W represent the standard deviation of PS values measured before sonication (N = 4).

The relationships between the PS product measured for each of the optical contrast agents and sonication power are shown in Fig. 3. For both dyes, there is a strong correlation between PS and power. However, because of the difference in molecular weight between the two dyes, their respective PS values at the same power differed greatly. Moreover, the mean baseline value determined for IRDye indicates there was some leakage across the intact BBB.

**Figure 4:**
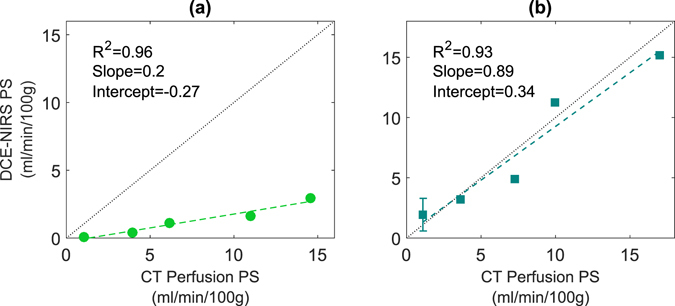
Regression analysis comparing PS values calculated for each of the optical dyes and the corresponding mean values determined by CT: (**a**) ICG data and (**b**) IRDye data. The dotted line in each graph represents the line of identity.

The individual correlation of PS values for each of the optical dyes and the corresponding values from CT are shown in Fig. 4. Similar to Fig. 3, a strong correlation with CT was found for both dyes. In each case the correlation slope was significantly different from zero; however, only the slope for the ICG-versus-CT comparison was statistically different from the line of identity. This again reflects the dependency of a contrast agent’s permeability on its molecular weight. That is, the leakage of ICG is considerably less compared to the two other contrast agents because it binds to blood proteins.

## Discussion

NIRS has become a well-established technique for measuring tissue saturation with a number of commercial systems marketed for monitoring brain health. Nevertheless, there is a continuing effort to develop optical methods that can more directly target key cerebral parameters such as CBF and cerebral oxidative metabolism^25, 26^. DCE NIRS is one such approach and has been previously used to detect CBF abnormalities in stroke patients^15, 27^ and in animal models of cerebral ischemia^16^. The work presented in this study extends the potential clinical utility of NIRS by presenting a DCE method of assessing BBB integrity. The approach requires minor modifications to existing DCE NIRS methods for assessing CBF. First, the NIRS system must be able to quantify *μ*_a_ in order to convert the time-varying changes in *μ*_a_ into a tissue concentration curve, although this criterion is the same for approaches used to quantify CBF^28–30^. Similarly, the arterial concentration curve must be measured, which can be done non-invasively by dye densitometry (Fig. 1a). Unlike CBF methods that only require less than a minute of data acquisition, assessing permeability requires longer acquisition times (~5 min) in order to capture the slow leakage of contrast agent into brain parenchyma. Finally, the most significant modification is with regards to analyzing the DCE data in order to characterize BBB leakage.

The high temporal resolution of NIRS – data were acquired every 200 ms in the current study – enabled the DCE data to be analyzed with a kinetic model capable of separating the effects of blood flow and permeability. This is in contrast to slower imaging modalities, notably magnetic resonance imaging, that typically can only estimate a single rate constant that lumps CBF and PS together^9^. For NIRS, the kinetic model was modified to include an additional fitting parameter to account for the distribution of possible vascular transit times in the relatively large tissue volume interrogated by the NIRS probes. Similar to our previous DCE NIRS study of tumor permeability^19^, this parameter was found to improve the fit of the model to the kinetic data. A potential trade-off with adding another fitting parameter is reduced precision in the parameter estimates. However, based on our previous error analysis^19^ and the high contrast-to-noise ratio of the DCE data (Fig. 1b and c), the uncertainties in the CBF and PS estimates were likely less than 5%.

Using FUS to open the BBB in these experiments enabled us to assess the sensitivity of DCE NIRS to changes in permeability since it has been shown that the leakage rate of a contrast agent is proportional to sonication power^31, 32^. This is clearly evident in Fig. 3, which shows a strong correlation between the measured PS values and sonication power. Furthermore, the slope of this correlation was different for the two optical dyes (ICG and IRDye) due to their different molecular weights. Likewise, a strong, statistically significant correlation with PS estimates from CT was found for both dyes. Similar to Fig. 3, the PS values for ICG were smaller than the corresponding CT measurements. In contrast, the slope of the correlation between PS values measured with IRDye and CT was close to 1, reflecting the similarity in size of these two contrast agents (1166 and 777 Da, respectively). These results demonstrate that DCE NIRS has the sensitivity to detect changes in BBB permeability and to provide accurate PS estimates given the agreement with independent measurements by CT.

An unexpected finding in this study was lower CBF after sonication as measured by the methods (i.e. NIRS and CT) (Table 1). It is uncertain if this perfusion decrease could be attributed to the FUS procedure, as a confounding factor was the delay of approximately 1 h between the two sets of DCE acquisitions, which was required to perform the FUS procedure. The effects of anesthetics and handling – each animal had to be moved from the CT bed to the FUS system for sonication – may have affected blood flow. As the primary focus of the study was on measuring the PS product and not CBF, these experiments did not include a control group to remove such confounders.

Considering that DCE NIRS with ICG has already been used to assess cerebral hemodynamics in patients^33^, the proposed approach in principle could be adapted to clinical studies. However, similar to other NIRS neuro-applications, using the proposed method to assess BBB integrity in critical-care patients will require careful investigation of the effects of signal contamination from extra-cerebral tissues given the inherently limited depth sensitivity of NIRS^34^. The impact of not accounting for scalp contamination was previously demonstrated in a study showing that DCE NIRS can substantially underestimate CBF in adults^35^. Methods to enhance depth sensitivity, in particular, time-resolved detection^11, 36^, have been developed and shown to benefit DCE NIRS applications in adults^10, 37^, including a study of patients with known BBB dysfunction^17^. Furthermore, the incorporation of optical reconstruction into the analysis of DCE NIRS has been shown to provide accurate CBF estimates in large animal models than mimic the typical thickness of the adult human head^16, 29^. An additional consideration with regards to measuring the PS product in the adult brain is the influence of possible contrast agent leakage into scalp tissue, which lacks the tight conjunctions between endothelium cells found in cerebral capillaries. Further studies using large animal models would be prudent to investigate this potential source of error. Such experiments would require a suitable FUS system (e.g. Exablate 4000, Insightec, Israel)^38^, which is beyond the current proof-of-principle study.

In summary, the results obtained during our study provide the basis for a simple, quantitative BBB evaluation method, which should allow for bedside monitoring of patients at high risk of BBB disruption^17^.

## Methods

### Animal Experiments

All experiments were approved by the Subcommittee of the Canadian Council on Animal Care (CCAC) and the Animal Use Committee at Western University (London, ON, Canada). All animal procedures described herein were carried out in accordance with the approved guidelines listed above. Male Wistar rats (480 ± 120 g) were used in the study. Prior to sonication, anesthesia was induced with 2.5% isoflurane using a nose cone, the head was shaved and depilated, and two tail vein catheters were inserted. Ketamine-xylazine (10:1) was then administered at a rate of 1–1.5 ml/h via one tail vein catheter and isofluorane was turned off. After 30 min, a probe holder was placed on the rat scalp, secured in place with velcro straps, and the optical probes were placed in the holder. The FUS procedure and the acquisition of the NIRS and CT data are described in the following sections. At the end of the experiment, animals were euthanized according to guidelines set forth by the CCAC.

### DCE NIRS Instrumentation and Measurements

**Figure 5:**
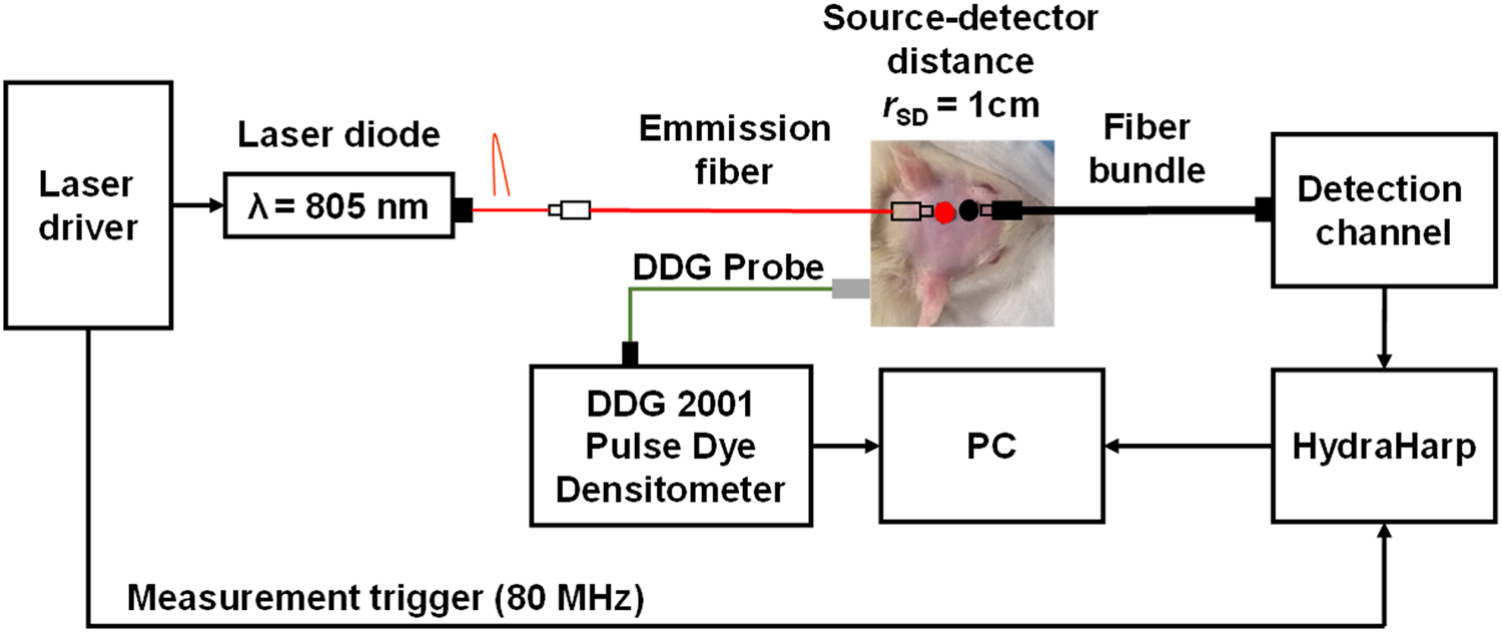
Schematic of the TR-NIRS system. Short pulses of light at 805 nm are generated (80 MHz) and guided to the rat’s head by an optical fiber. Another fiber bundle directs diffusively reflected light from the head to fast detectors coupled to a counting board (HydraHarp).

The time-resolved system (see Fig. 5), which has been described in detail elsewhere^39, 40^, used one semiconductor diode laser (LDH-P-C-810, PicoQuant, Germany) to emit picoseconds light pulses at a frequency of 80 MHz and a wavelength 805 nm, which is close to the maximum absorption wavelength of ICG and IRDye. The laser pulses were coupled from the laser head into an emission fiber (*ϕ* = 400 μm, NA = 0.22, Fiberoptics Technology, Pomfret, CT, United States). Diffusely reflected light was collected with a 2 m long fiber bundle (*ϕ* = 3 mm, Fiberoptics Technology, Pomfret, CT, United States) coupled to a hybrid photomultiplier detector (PMA Hybrid, PicoQuant, Germany) and a time-correlated single photon counting module (HydraHarp 400, PicoQuant, Germany). An in-house 3D-printed (BFB-3000, 3D Systems) holder was used to position the source fiber and detection fiber bundle on the surface of the rat’s head over the left cerebral hemisphere at a source-detector distance (*r*_SD_) of 1 cm. The TR system was mounted in a portable cart for transport to the CT suite.

For each experiment, two sets of DCE NIRS and CT data were collected prior to and after sonication. One of four sonication powers was selected for each experiment (see next section). To avoid possible contamination between clearance curves for the two optical dyes, only one dye was used in a given experiment. The DCE NIRS protocol consisted of a rapid bolus injection of the dye (iv, 0.1 mg/kg), followed by serial NIRS acquisition at intervals of 200 ms for a total of 480 s. Concurrently, the arterial concentration of the optical dye was measured non-invasively by a dye densitometry attached to the back paw.

### BBB opening procedure

**Figure 6:**
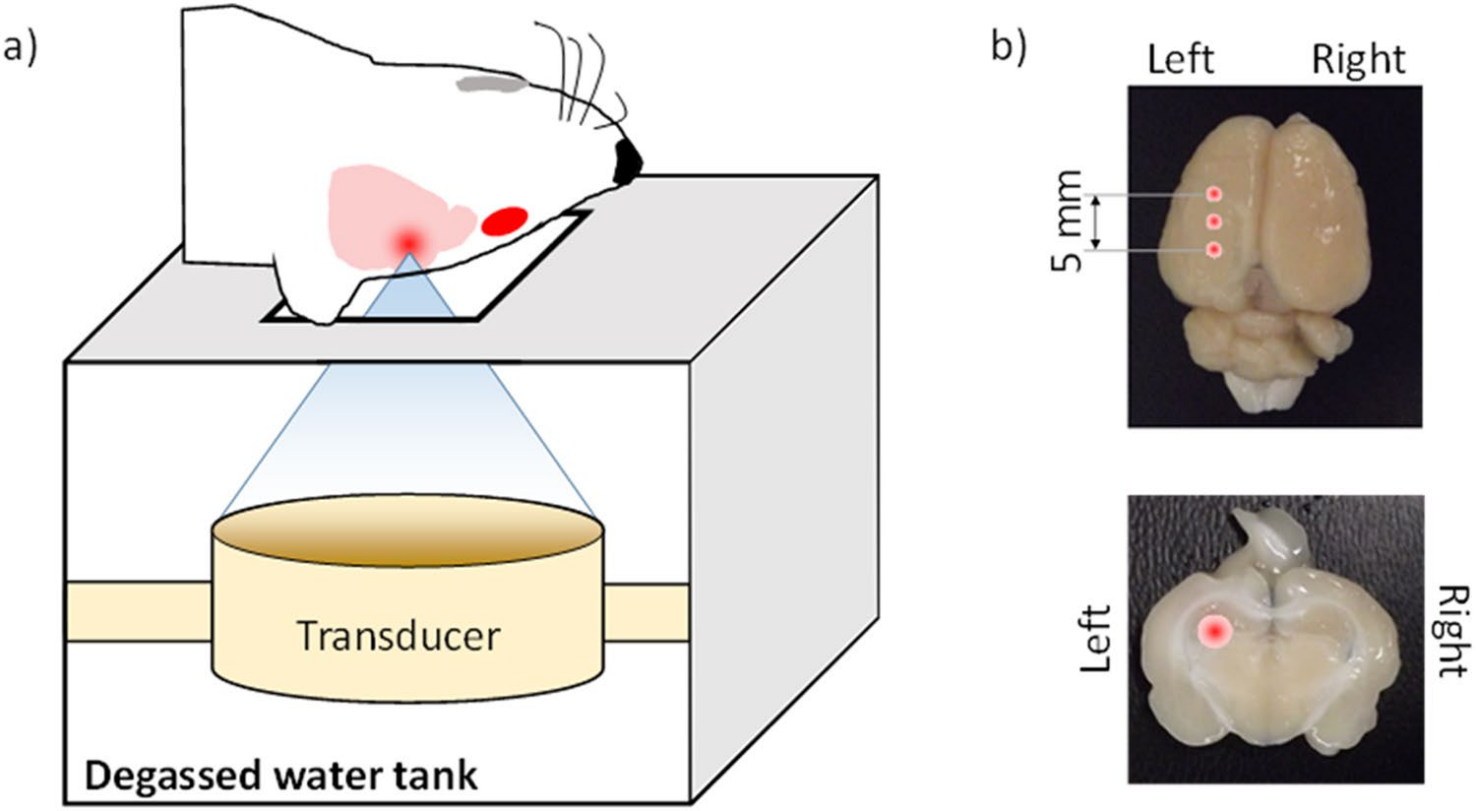
The FUS system was placed on the bed of a Revolution CT scanner while (**a**) rat’s head was placed over the transducer. (**b**) Locations of sonication points displayed on an excised rat’s brain.

Opening the BBB was performed by computer‐controlled image-guided focused ultrasound (FUS) (RK‐100, FUS instruments Inc., Toronto, ON). The system was equipped with a spherically focused transducer (7.5 cm diameter, f# = 0.8) designed for operating at frequencies between 0.25 and 1.0 MHz and acoustic powers ≤50 W (continuous power). For image guidance, the FUS system was co-registered to the spatial coordinates of the CT scanner. Next, the rat was placed supine with its head above the ultrasound transducer (Fig. 6a), which was mounted inside a tank filled with degassed water. Each sonication was performed with a frequency of 0.563 MHz, a pulse rate of 1 Hz and a burst length of 10 ms. The procedure for BBB opening required an intravenous injection of Definity microbubbles (0.02 mL/kg, Lantheus Medical Imaging, MA), followed by 120 s of sonication. In each experiment, the sonication power was selected from one of four values (0.5, 1.0, 1.5 and 2 W) to vary the degree of BBB opening. Sonication was performed on the left hemisphere of the brain at three locations separated by 5 mm in order to cover the sensitivity volume of the NIRS probes (Fig. 6b).

Computed tomography anatomical images and dynamic data for mapping BBB permeability were acquired using a Revolution scanner (GE Healthcare, Waukesha, WI). Dynamic CT data were acquired during the bolus injection of an iodine-based low molecular weight (777 Da) contrast agent (Isovue^®^-300, Bracco Diagnostics Inc., Vaughan, Canada, 300 mg iodine/ml, 2.5 ml/kg body weight) at a rate of 0.13 ml/s (slice thickness = 1.25 mm, current = 200 mA, energy = 80 kVp, DFOV = 250 mm).

### Data analysis

BBB permeability was characterized in terms of the PS product, which was derived by fitting a tracer kinetic model to ICG and IRDye clearance curves. This model has been used previously to assess vascular permeability of the same contrast agents in tumors^19^. In the current study, the time varying change in the absorption coefficient, *μ*_a_(t), due to passage of contrast agent through brain was calculated from the change in area of the measured DTOFs. The *μ*_a_(t) data set was then converted into a tissue concentration curve, *C*_tis_(t), using the appropriate extinction coefficient of the injected dye. Similarly, the arterial time-varying absorption data acquired by dye densitometry were converted into an arterial concentration curve, *C*_art_(t), using the dye’s extinction coefficient and the measured total hemoglobin concentration, which was determined by acquiring a blood sample from each rat.

The time-dependent tissue and arterial blood concentration curves can be related by the following expression:

1 $${C}_{tis}(t)=CBF\cdot {\int }_{0}^{t}{C}_{art}(u)\cdot R(t-u)du$$

where: CBF is cerebral blood flow and *R*(t) is the impulse residue function, which represents the fraction of contrast agent that remains in the tissue volume at time *t* for an idealized bolus injection at *t* = 0 of unit concentration. *R*(t) was defined by the adiabatic approximation to the tissue homogeneity (AATH) model, which has been used in DCE MRI and CT experiments^41^. It accounts for the fraction of contrast agent that leaks into the interstitial space (*E*), the fraction remaining in blood (1-*E*), and the clearance rate of contrast agent from tissue (*k*_e_). For NIRS, the model was modified to account for a distribution of capillary transit times since the NIRS probes interrogate a relatively large brain volume (of the order of 1 cm^3^)^19^:

2 $$R(t)=1-{\int }_{0}^{t}g(u)du+E\cdot {e}^{-{k}_{e}t}{\int }_{0}^{t}g(u){e}^{{k}_{e}u}du$$

where, *g*(t) is a gamma distribution of capillary transit times with width *α* ^−1^. In total, *R*(t) is described by five parameters: *CBF*, *E*, *k*_e_, a mean capillary transit time, and *α* ^−1^.

A nonlinear optimization routine MATLAB^®^ function (fminsearch) was used to fit the model to *C*_tis_(*t*) using equation (1) in order to extract best-fit estimates of the five parameters. The PS product was calculated from the estimates of CBF and E by:

3 $$PS=-\,CBF\cdot \,\mathrm{ln}\,(1-E)$$

The DCE CT data were analyzed using CT Perfusion 5 software (GE Healthcare), which generates functional maps including for CBF and the PS product. Region-of-interest (ROI) analysis was performed for comparison to the NIRS results. The ROI was based on Monte-Carlo simulations of light propagation through a homogeneous medium with optical properties reported for the rat brain^42^ (*μ*_a_ = 0.2 cm^−1^ and a reduced scattering coefficient of 7.5 cm^−1^) and a source-detector separation of 1 cm, corresponding to the distance used in the experiments. The shape and size of the ROI was defined by only including photon pathlengths that were within 3% of the maximum value of the simulated DTOF. These thresholds were the same as used in the area-under-the-curve analysis of the DTOFs measured in the DCE experiments. Based on a source-detector separation of 1 cm, the volume of tissue interrogated by the NIRS probe was approximately 0.4 cm^3^. The ROI was applied to the functional images and mean values of CBF and the PS product were extracted.

### Statistical analysis

For each optical dye, correlations between DCE NIRS and CT PS values were assessed by linear regression. Likewise, linear regression was used to correlate DCE NIRS PS values to sonication power. T-tests were performed to compare mean correlations slopes to the null hypothesis (i.e. slope = 0) and the line of identity (a slope of 1). For the analysis of the CBF results, the measurements for the two optical dyes and across the four sonication powers were grouped together since CBF was expected to be independent of both variables. A two-way analysis of variance ANOVA was conducted to assess interactions between CBF and technique (NIRS and CT) and time (pre and post sonication). All statistical analyses were conducted using SPSS 16.0 (SPSS, Chicago, Illinois) with a statistical significance level of p < 0.05.
